# Basis-neutral Hilbert-space analyzers

**DOI:** 10.1038/srep44995

**Published:** 2017-03-27

**Authors:** Lane Martin, Davood Mardani, H. Esat Kondakci, Walker D. Larson, Soroush Shabahang, Ali K. Jahromi, Tanya Malhotra, A. Nick Vamivakas, George K. Atia, Ayman F. Abouraddy

**Affiliations:** 1 CREOL, The College of Optics & Photonics, University of Central Florida, Orlando, FL 32816, USA; 2 Department of Electrical and Computer Engineering, University of Central Florida, Orlando, FL 32816, USA; 3 Department of Physics and Astronomy, University of Rochester, Rochester, New York 14627, USA; 4 Center for Coherence and Quantum Optics, University of Rochester, Rochester, New York 14627, USA; 5 Institute of Optics, University of Rochester, Rochester, NY 14627, USA

## Abstract

Interferometry is one of the central organizing principles of optics. Key to interferometry is the concept of optical delay, which facilitates spectral analysis in terms of time-harmonics. In contrast, when analyzing a beam in a Hilbert space spanned by spatial modes – a critical task for spatial-mode multiplexing and quantum communication – basis-specific principles are invoked that are altogether distinct from that of ‘delay’. Here, we extend the traditional concept of *temporal* delay to the *spatial* domain, thereby enabling the analysis of a beam in an arbitrary spatial-mode basis – exemplified using Hermite-Gaussian and radial Laguerre-Gaussian modes. Such generalized delays correspond to optical implementations of fractional transforms; for example, the fractional Hankel transform is the generalized delay associated with the space of Laguerre-Gaussian modes, and an interferometer incorporating such a ‘delay’ obtains modal weights in the associated Hilbert space. By implementing an inherently stable, reconfigurable spatial-light-modulator-based polarization-interferometer, we have constructed a ‘Hilbert-space analyzer’ capable of projecting optical beams onto any modal basis.

Interferometry is the cornerstone of fundamental investigations and precise measurements in optics^1^. The nature of light – both classical^2^,^3^ and quantum^4^,^5^,^6^ – was unraveled largely through interferometric experiments, and the exquisite precision inherent in optical interferometry has been instrumental in metrology^7^, bio-imaging^8^, devising ultra-sensitive systems for the detection of gravitational waves^9^, and enabling novel lithographic schemes^10^. These examples share a common feature: interference results from combining beams with relative phases engendered by optical delays. A principal utility for optical interferometry is spectral analysis – determining the contributions of the *continuum* of time-frequency harmonics to the optical signal. Recent applications have emphasized the utility of *discrete* spatial-mode bases for optical beams, such as orbital angular momentum (OAM) states^11^,^12^,^13^ exploited in free-space^14^,^15^ and multimode fibers^16^,^17^ to increase their information-carrying capacity (so-called spatial-mode multiplexing) and in quantum communication protocols^18^ (such as quantum key distribution^19^). An optical beam in this conception is an element in a Hilbert space spanned by such a basis. In general, strategies for spatial-mode analysis rely on approaches altogether different from the concept of optical delays that has served interferometry so well. In other words, we currently lack a ‘Hilbert-space analyzer’: a hypothetical device capable of analyzing an optical beam in the vector space defined by any prescribed modal basis. Examples of strategies for modal analysis range from phase-retrieval combined with direct mode projections^20^, correlating the modes with spectral or temporal degrees of freedom^21^, combining principal-component analysis after adapting the detection system with a training data set^22^, to performing a coordinate transformation that converts the beam into a more convenient basis^23^. In particular, despite multiple techniques for OAM beam analysis^24^,^25^,^26^,^27^, comparable progress has been lacking for other important modal bases, such as radial Laguerre-Gaussian^28^,^29^,^30^,^31^,^32^ (LG) modes.

In archetypical two-path interferometers, two copies of a beam are combined after a relative optical delay is inserted. The delay is swept and an interferogram is traced, which yields the modal weights of time-frequency harmonics through spectral analysis. In this paper, we present a unifying principle for modal analysis by addressing the following question: can the traditional optical delay – one of the most fundamental concepts in optics – be extended beyond its implementation in the time domain to apply to Hilbert spaces associated with discrete spatial-mode bases? We show here that such a generalization is indeed possible. We introduce the concept of a *generalized delay* (GD): an optical transformation characterized by a continuous, real order-parameter that can be tuned to produce – once placed in one arm of an interferometer – an interferogram that reveals the modal weights in a prescribed functional basis via harmonic analysis. We find that GDs correspond to optical implementations of fractional transforms in the case of discrete modal bases^33^,^34^. For example, it can be shown^34^ that the GD associated with Hermite Gaussian (HG) modes is the fractional Fourier transform^35^,^36^, whereas that associated with radial LG modes^37^ is the fractional Hankel transform^38^,^39^. Sweeping the order of a fractional transform corresponds to varying a temporal delay in traditional interferometry – each in its own Hilbert space.

In the implementation presented here, we exploit electrically addressable spatial light modulators (SLMs) to realize tunable-strength cylindrical and spherical lenses that are building blocks of fractional transforms^40^. We make use of the polarization discrimination of SLMs^41^,^42^ to construct a polarization interferometer – in lieu of a two-path interferometer – to accomplish generalized interferometry in an inherently stable configuration. Switching between Hilbert spaces – that is, examining a beam in different bases – is readily achieved in the same setup with no moving parts, simply by changing the phases imparted by the SLMs. We thus establish a versatile, basis-neutral Hilbert-space analyzer based on a generalized conception of optical interferometry.

## Concept of a generalized optical delay

**Figure 1:**
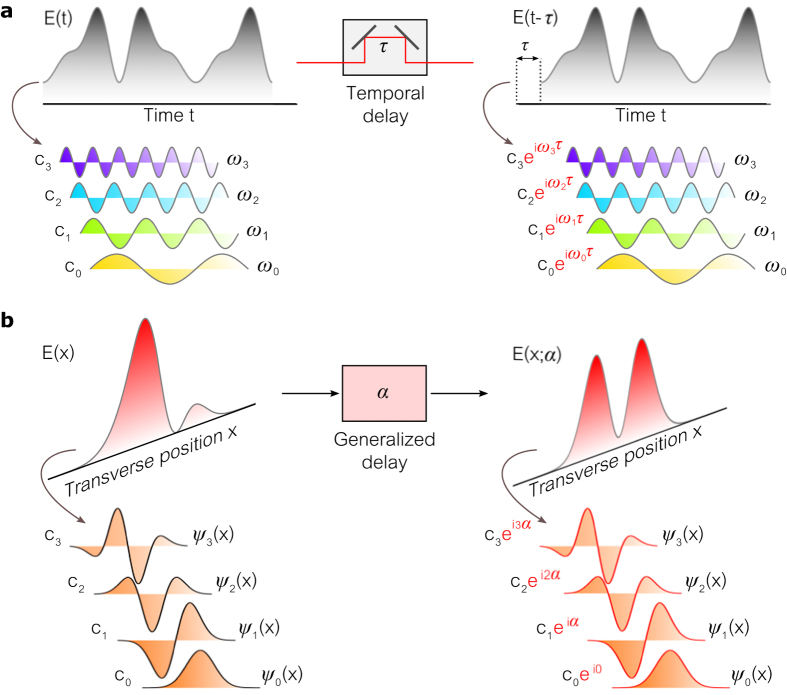
Concept of a generalized optical delay. (**a**) Traditional temporal optical delay. The impact of a temporal delay *τ* on a pulse *E(t*) can be viewed in two ways. In the time domain (first row), the pulse is delayed, *E(t* − *τ*). In the spectral domain (second row), the pulse is a superposition of temporal harmonics 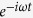 (angular frequencies *ω*) each with a spectral amplitude *c*_*n*_. The delayed pulse *E(t* − *τ*) is the result of inserting phase factors 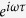 for each harmonic *ω*. (**b**) Generalized delay (GD) *α* in a Hilbert space spanned by a discrete modal basis 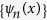. The impact of the GD on an optical beam can also be viewed in two domains. In the spatial domain (first row), the GD is not simply a shift but instead it transforms the transverse field profile *E(x*) → *E(x*; *α*). However, in the modal space (second row) where the field is viewed as a superposition of the modes 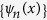 with weights 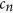, the impact of the GD is identical to that of the temporal delay on the spectral harmonics in (**a**). The GD adds a phase factor 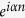 to the 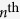 mode amplitude, which ‘delays’ the beam by *α* in the Hilbert space spanned by 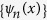.

An optical delay *τ* is typically implemented by inserting an additional propagation length in a beam’s path. In the time domain, a delay shifts the temporal origin *E(t*) → *E(t* − *τ*), whereas in the spectral domain it adds to each harmonic frequency component *ω* a phase *e*^*iωτ*^ that is linear in both the delay and the frequency (Fig. 1a). In other words, spectral harmonics {*e*^−*iωt*^} are eigenstates of the delay operation with eigenvalues *e*^*iωτ*^. Guided by this observation, we introduce a generalized delay (GD) that operates in the Hilbert space spanned by a modal basis {*ψ*_*n*_(*x*)}, such that the GD’s effect on a beam described in this space is completely analogous to that of a delay *τ* for a pulse. A GD operates between an input plane 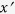 and output plane *x*, and implements a unitary transformation 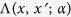 characterized by a real, continuous order-parameter *α*,


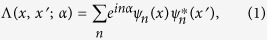


where the functional basis 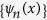 is orthonormal and complete, and its members are the eigenstates of Λ: they emerge from the GD unchanged except for a mode-dependent phase 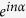 (Fig. 1b), 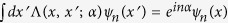; see Methods.

Consider a monochromatic beam 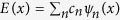, where 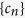 are modal coefficients and *E(x*) is normalized 
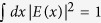
, such that 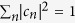. Upon passage through the GD, the field is transformed according to


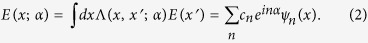


Each mode thus acquires a phase 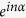 that depends linearly on its index *n* (Fig. 1b) – in analogy to the impact of a traditional delay with respect to spectral harmonics. For a discrete modal basis indexed by *n* (Equation 1), 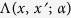 is periodic in *α* with period 2*π*. Furthermore, Λ can be generalized to two transverse coordinates and is applicable to a continuous basis^33^,^34^.

As an example, consider the set of one-dimensional (1D) HG modes, 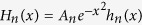, where 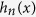 is the *n*^th^-order Hermite polynomial and 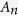 is a normalization constant. This modal set is well-established as a useful basis for laser beams and arises naturally in many contexts^43^. The corresponding GD is the 1D fractional Fourier transform (fFT)^34^ of angular-order *α* (scaled heretofore by convention from 0 to 4). Indeed, HG modes are eigenstates of the fFT^36^ with eigenvalues 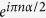. A beam traversing this GD is not shifted in *physical* space, as an optical delay shifts a pulse in time. Nevertheless, because each underlying HG mode acquires the requisite phase after the GD, the fFT ‘delays’ the beam *in the Hilbert space of optical beams spanned by HG modes*, which thus facilitates analyzing the beam in the HG basis. Alternatively the set of *radial* LG modes associated with zero-OAM states given by 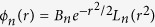 constitutes a modal basis for radial functions having azimuthal symmetry; here 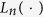 is the *n*^th^-order Laguerre polynomial, 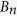 is a normalization constant, and *r* is a radial coordinate. The GD here corresponds to the fractional Hankel transform (fHT); i.e., an optical implementation of the fHT ‘delays’ the beam in the Hilbert space spanned by radial LG modes^33^. Techniques for beam analysis into radial LG modes are lacking, leading the radial coordinate to be recently dubbed the ‘forgotten’ degree of freedom^29^.

## Generalized optical interferometry

**Figure 2:**
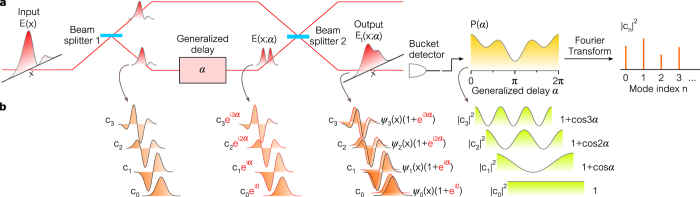
Generalized optical interferometry for modal analysis in an arbitrary basis. (**a**) Operation of a generalized interferometer in real space. Two copies of the beam *E(x*) are created at beam splitter 1 and subsequently combined at beam splitter 2 after one copy traverses the GD and is ‘delayed’ in the associated Hilbert space by *α, E(x*; *α*). The beam emerging from the interferometer – a superposition of the delayed beam and a reference 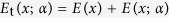 – is collected by a bucket detector and an interferogram is recorded with *α*, 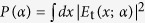, whose Fourier transform reveals the modal weights 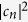. (**b**) Operation of the generalized interferometer in the Hilbert space spanned by the modal basis 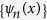 on the beam 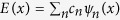 (Fig. 1b). The underlying modes of the ‘delayed’ copy acquire phase shifts of the form 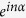 after passing through the GD to yield a new beam 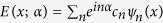. The original and ‘delayed’ beams are combined 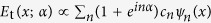 to produce an interferogram 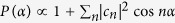. Because the modes are orthogonal to each other, each interferes only with its phase-shifted counterpart to yield an interferogram of the form 1 + cos *nα* with weights 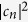 – independently of the underlying basis 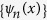 that is traced out at the bucket detector. The sought-after weights are then revealed through harmonic analysis.

A GD can be exploited for the modal decomposition of an optical beam in its associated Hilbert space. The overall scheme for ‘generalized optical interferometry’ is a balanced two-path interferometer, in which the usual temporal delay is replaced by a GD (Fig. 2a). For an incident beam 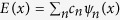 and a GD constructed using the modal basis 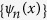, the output field is 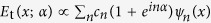 and the power recorded by a ‘bucket detector’ is


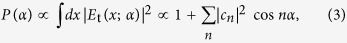


such that harmonic analysis of *P(α*) identifies the weights 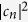; Fig. 2b. Each mode thus produces *individually* a sinusoidal interferogram ∝1 + cos *nα*. Mode-orthogonality dictates that each mode interferes only with itself. Crucially, the form of the interferogram in Eq. 3 is *independent* of the particular modal basis. A superposition of two HG modes of order *n* and *m*, for example, yields an interferogram that is identical to the same superposition of LG modes of order *n* and *m* – if the appropriate GD associated with each Hilbert space is implemented. This generalized interferometer is thus ‘basis-neutral’. Furthermore, since the GD associated with a discrete modal basis is periodic in its order *α*, the resulting interferogram is in turn periodic, such that its Fourier transform yields a discrete spectrum. The number of modes that may be distinguished in this manner is determined by the sampling rate of the interferogram (the number of settings of *α* measured) and is ultimately Nyquist-limited.

## Experimental implementation

A fFT or fHT can be implemented via combinations of cylindrical or spherical lenses, respectively, and the transform orders are varied by changing either the lens strengths or their separation (or both)^36^,^44^. The former approach does not require moving parts and can be realized with electrically addressable phase-only SLMs that implement generalized lenses of variable power – which is the strategy we follow here. A minimum of three generalized lenses can implement a 1D fFT^40^, where the first and last lenses have the same power and the distances separating the SLMs are equal (Methods). The fFT order can thus be varied *without* overall scaling or additional phases imparted to the field^40^, which is critical since we will interfere the beam with its own fFT.

**Figure 3:**
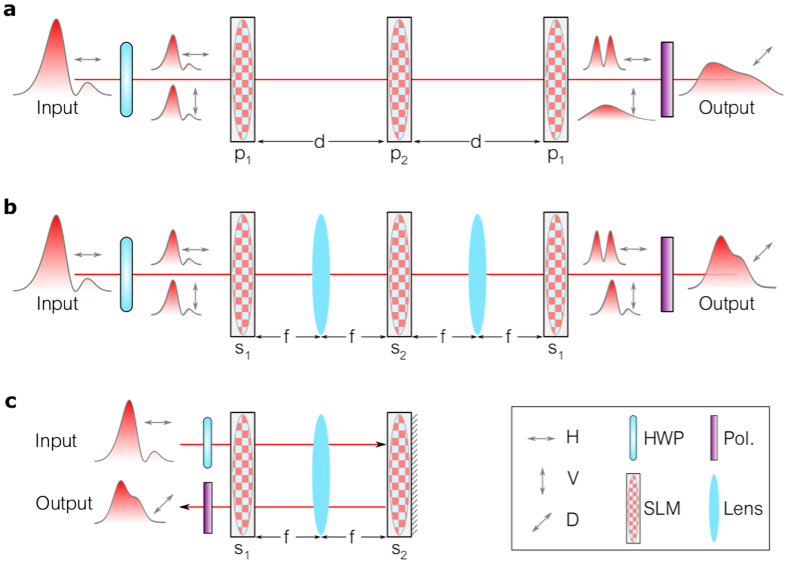
Inherently stable implementation of a generalized interferometer. (**a**) Implementation of a 1D fFT using three generalized (variable-power) lenses L_1_, L_2_, and L_3_ with symmetric strengths *p*_1_, *p*_2_, and *p*_1_, respectively, that are selected to produce a fractional transform of prescribed order (Methods). Because the lenses are implemented by polarization-selective SLMs (affecting only the H-component), the system is in fact equivalent to the two-path interferometer in Fig. 2a, with the H- and V-components corresponding to the delay and reference arms, respectively, while the half-wave plate (HWP) and the polarizer correspond to beam splitters 1 and 2, respectively. This common-path interferometer is inherently stable. However, the V-component undergoes unwanted diffraction over the distance 2*d*. (**b**) Same as (**a**), except that polarization-insensitive fixed lenses (focal lengths *f*) are inserted in a 4 *f* configuration to eliminate the diffraction of the V-component. The strengths *s*_1_, *s*_2_, and *s*_1_ of the generalized lenses are modified to compensate for the added lenses. (**c**) Folded implementation of (**b**). The beam is reflected onto itself from L_2_, such that L_1_ and L_3_ are the same generalized lens and only one fixed lens is required.

The two-path interferometer in Fig. 2a requires a high degree of stability since several large components (SLMs) are introduced into one path, the overall path lengths may be large (~1 m here), and a fractional-transform-order-dependent relative phase must be included (Methods). These difficulties are obviated by introducing a novel configuration that exploits the polarization-selectivity of liquid-crystal-based SLMs^41^ to construct the *single-path* polarization interferometer (Fig. 3a). The three SLMs impact the horizontal polarization component H, whereas the vertical component V is unaffected. After rotating the input polarization to 45°, only the H-component is transformed by the SLMs whereas the V-component is unchanged, thus serving as a reference. Projecting the output polarization at 45° allows the H and V components to interfere. However, the V-component undergoes diffraction during propagation and at the output it no longer corresponds to the original field *E(x*) needed as a reference. We therefore introduce lenses between the SLMs arranged in a 4-*f* configuration to image the V-component and reproduce *E(x*), and modify the strength of the lenses implemented by the SLMs accordingly (Fig. 3b; Methods). Since the symmetry of the configuration is maintained, reflective SLMs allow folding the system such that only two SLMs and one lens are required (Fig. 3c). This stable polarization interferometer is thus in one-to-one correspondence with the two-path interferometer in Fig. 2a.

In implementing the fHT, we require that the SLMs produce simultaneously equal-order1D fFTs along *x* and *y*. Each SLM thus corresponds to equal-power crossed cylindrical lenses, or a spherical lens.

## Results

We first realize modal analysis via generalized interferometry in the basis of 1D HG modes, where the associated GD is the 1D fFT. We examine beams having the separable form 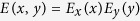 and focus on the *x*-dependence alone. The input beams are prepared by a single SLM (SLM_0_) that imprints a phase-only pattern on a Gaussian-mode laser beam, which is then imaged to SLM_1_ that constitutes the input plane to the generalized interferometer. A second SLM (SLM_2_) reflects the beam back to SLM_1_, and the phases imparted by SLM_1_ and SLM_2_ are varied to cycle the fFT order *α*.

**Figure 4:**
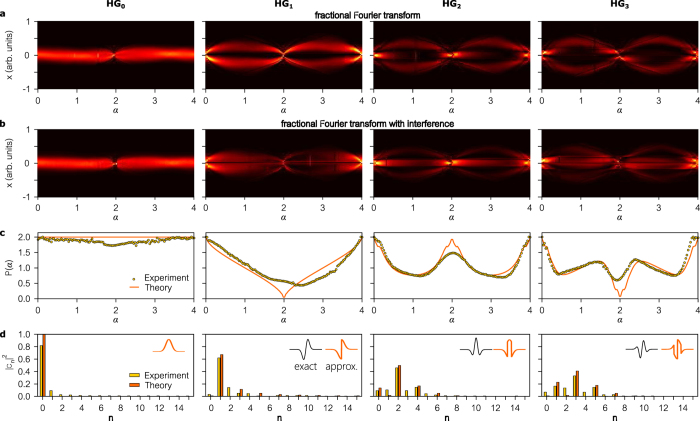
Modal analysis in the Hilbert space spanned by 1D Hermite-Gaussian modes using generalized optical interferometry. (**a**) The measured ‘delayed’ beam resulting from the input beam *E(x*) (which is to be analyzed into the contributions from HG modes) traversing the order-*α* GD (here the fFT), 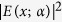. Each vertical line plot represents the magnitude-squared of a 1D fFT 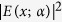 associated with a different order *α*. (**b**) The measured interferogram resulting from superposing the delayed beam from (**a**) with a reference, 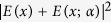. Each vertical line plot thus represents the magnitude-squared of the 1D spatial interferogram associated with a different order *α*. (**c)** The integrated interferogram 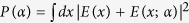. This interferogram is now basis-neutral. (**d**) The modal weights |*c*_*n*_|^2^ revealed by taking the Fourier transform of the interferogram in (**c**). The columns are for different input beams corresponding to modes HG_0_ through HG_3_. The implemented beams only approximate the pure HG modes (except for HG_0_ which is exact), as shown in the insets in (**d**). The black mode profile in the inset is an exact HG mode while the orange plot is the approximate beam used in the experiment. The theory plots in (**c**) and (**d**) are those for the implemented approximate beams. See Supplementary Information for theory.

We report in Fig. 4 measurements carried out on 1D beams approximating the four lowest-order HG modes. For each beam, we provide: (1) the intensity of the ‘delayed’ beam after the fFT 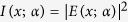 while varying the ‘delay’ *α*; (2) the intensity after interfering the delayed beam with the original, 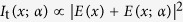; (3) the interferogram recorded by the ‘bucket detector’ 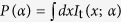; and (4) the Fourier transform of *P(α*) that reveals the modal weights 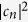. In Fig. 4a, each vertical line plot corresponds to the magnitude squared of a 1D fFT 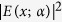 associated with a different order *α*, whereas each vertical plot in Fig. 4b is the corresponding spatial interferogram 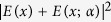. These data enable us to diagnose the system and evaluate its performance, but only the interferogram *P(α*) is required for modal analysis, which corresponds to the temporal interferogram obtained in traditional two-path interferometers incorporating an optical delay. This interferogram is basis-neutral now that the spatial degree of freedom has been integrated over.

Whenever *E(x*) is a pure *n*^th^-order HG mode, the interferogram 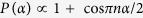 is a sinusoid whose Fourier transform produces a delta function at *n*. We verify this with modes 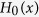 through 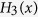. Because the Gaussian beam 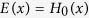 is an eigenstate of the fFT, we do not observe modulation in 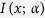 or 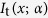, and the interferogram *P(α*) is thus a constant whose Fourier transform has a single contribution at *n* = 0. Next, the 1^st^-order HG mode 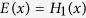 produces an interferogram having a full sinusoidal period *P(α*) ∝ 1 + cos *α* whose Fourier transform reveals the strongest contribution at *n* = 1. We approximate 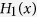 by imparting a *π*-phase step (via SLM_0_) to a Gaussian beam (Supplementary Information Sec. S3), so contributions from other modes appear in the modal analysis, and simulations provide a computed modal content that is in excellent agreement with the measurements (Supplementary Information Sec. S4). Similarly, 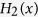 and 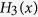 produce shorter period sinusoids and reveal the strongest contributions at *n* = 2 and *n* = 3, respectively. We note a discrepancy at the fFT order *α* = 2, whereupon the rapid variation imposed on the SLM phases results in a sudden drop in diffraction efficiency (Supplementary Information Sec. S5).

**Figure 5:**
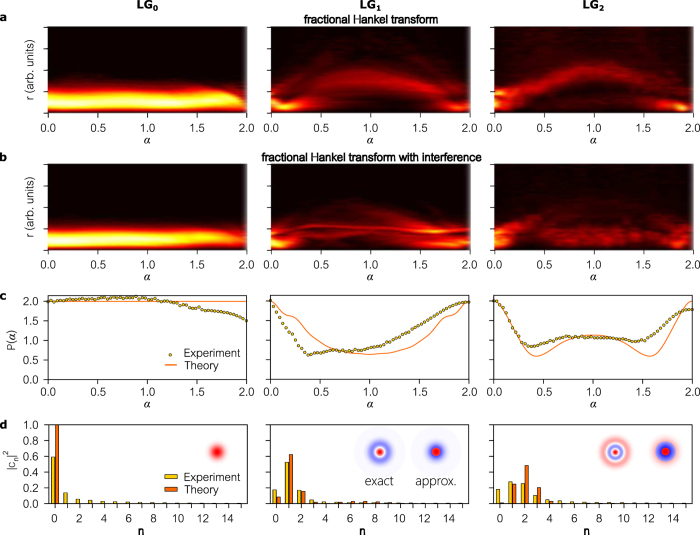
Modal analysis in the Hilbert space spanned by radial Laguerre-Gaussian modes using generalized optical interferometry. (**a–d**) Same as (**a–d**) in Fig. 4 except that the GD operates in the space of radial LG modes. Note that in (**a**) and (**b**), the delayed beam and the interferogram are plotted with *r* and not *x* (0 ≤ *r* ≤ ∞). Insets show the radial intensity distribution of the beams. The columns are for different input beams corresponding to modes LG_0_ through LG_2_. The implemented beams only approximate the pure radial LG modes (except for LG_0_ which is exact), as shown in the insets in (**d**). The mode profile on the left in the inset is an exact LG mode while the plot on the right is the approximate beam used in the experiment. The theory plots in (**c**) and (**d**) are those for the implemented approximate beams. See Supplementary Information for theory.

We next analyze beams into radial LG modes by implementing the fHT as the GD. The results for 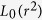 through 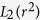 are presented in Fig. 5. Since these beams are azimuthally invariant, we first integrate the recorded 2D intensity *I(r, θ*) in polar coordinates over *θ* to obtain a 1D radial distribution 
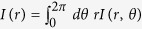
, where *I(r*) is the power in a thin annulus of radius *r* centered on the beam axis. Figure 5a depicts the ‘delayed’ beam *I(r*; *α*) as we vary the fHT-order *α*. Integrating over *r* after interfering the delayed beam with the reference produces the interferogram *P(α*). The basis-neutrality is clear when comparing the interferograms associated with 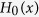 in Fig. 4 to 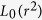 in Fig. 5; similarly for 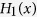 and 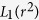, and for 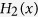 and 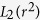.

**Figure 6:**
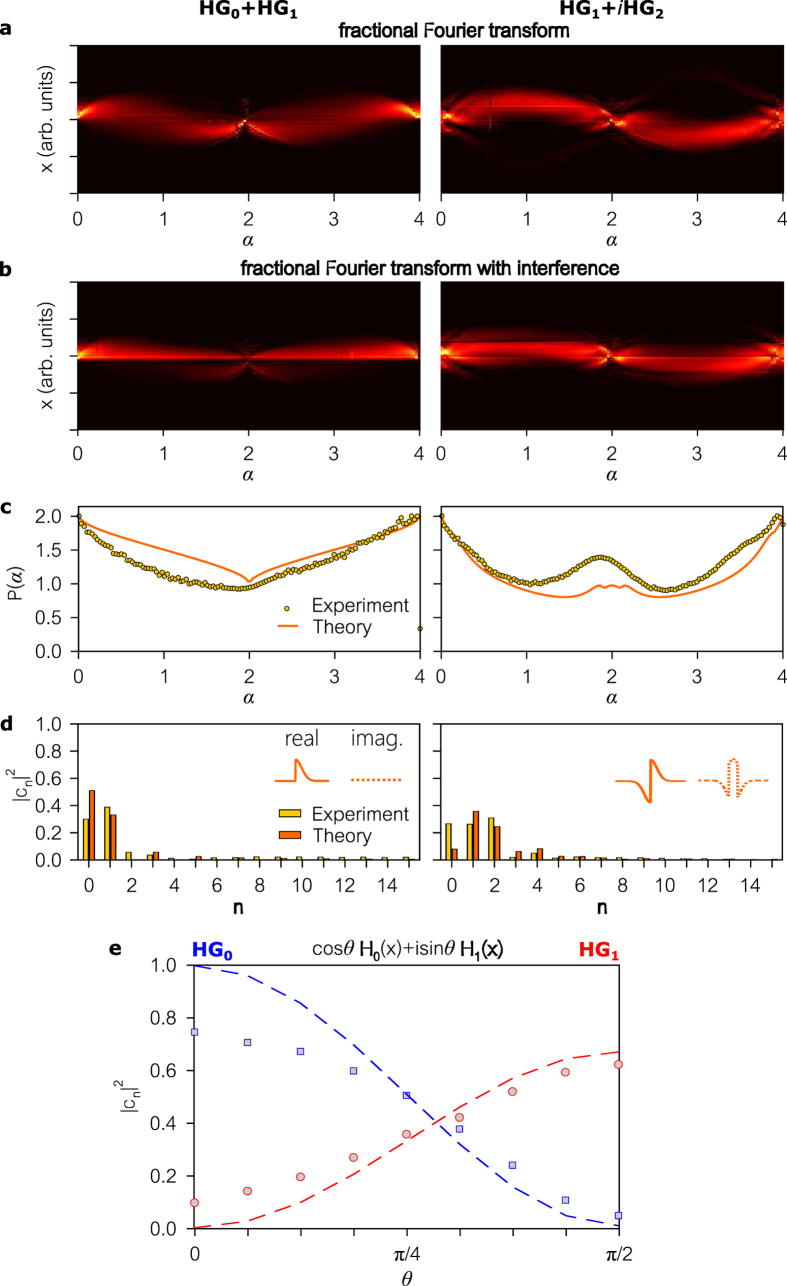
Modal analysis of beams comprising superimposed modes. (**a–d**) Same as (**a–d**) in Figs 4 and 5. The Input beams are the superpositions 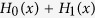 (left column) and 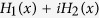 (right column). (**e**) Modal analysis of the beam 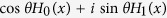, while varying *θ* from 0 to *π*/2. Plotted are the coefficients |*c*_0_|^2^ (blue squares) and |*c*_1_|^2^ (red circles), corresponding to the contributions of the modes HG_0_ and HG_1_. Dashed curves are theoretical predictions, for |*c*_0_|^2^ and |*c*_1_|^2^ predicated on the generated approximate modes.

To highlight the versatility of this approach, we examine beams formed of various superpositions of HG modes in Fig. 6. First, we analyze the beam 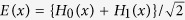 which we approximate by blocking half the cross section of a Gaussian beam (Fig. 6a–d). Next, we examine the field 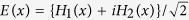 which we approximate by only varying the phase of a Gaussian beam to maximize the overlap with the desired beam (Fig. 6a–d). Finally, we investigate the superposition 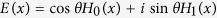 while varying *θ* from 0 to *π*/2, thereby switching the beam from 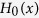 to 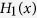 (Fig. 6e).

## Discussion and Conclusion

We have demonstrated that optical interferometry can be generalized to apply for any modal basis by replacing the traditional temporal delay with a generalized delay (GD): an optical transformation that ‘delays’ the beam in a Hilbert space spanned by the modal basis of interest. This basis-neutral strategy provides a unifying framework for modal analysis in an arbitrary basis – whether discrete, continuous, or combinations thereof for different degrees of freedom^34^. The fFT performs a rotation of the Wigner distribution associated with the field^45^, which has been exploited in tomographically reconstructing the Wigner distribution of non-classical states of light^46^. We have implemented this strategy here in the spatial domain of a scalar field using monochromatic light, but the approach is readily extended to multiple degrees of freedom of the optical field by simply cascading the associated GDs^34^. This methodology is also applicable to quantum states of light, such as one-photon or even entangled two-photon states^47^ by replacing the dual delays in a phase-unlocked HOM interferometer^48^ with the appropriate GDs. Our approach can thus further increase the accessible dimensionality of the Hilbert space of single photons by at least an order of magnitude^49^,^50^.

The accessible dimension of the beam’s Hilbert space is ultimately limited by the spatial resolution of the SLM pixels and the phase-step resolution for each pixel, which limit the sampling resolution of the fractional-transform order. Improvements in SLM technology may allow for real-time modal analysis over large-dimensional Hilbert spaces. One can use instead amplitude-based spatial modulators which are considerably faster, resulting in real-time modal analysis, albeit at the price of reduction in signal throughput^51^. We have found however that the physical extent of the SLM (or the *number* of pixels) is the main factor that limits the fidelity of modal analysis (see Supplementary Information for a detailed study).

Many new questions are now open: What is the optimal implementation of a GD when only a closed subspace of the modal basis is of interest? What is the minimum number of SLMs required to implement a GD in an arbitrary modal basis? Moreover, it is usually the case that only a few modes are activated (such as in communications protocols) or contribute significant energy – so-called modal ‘sparsity’^52^. In these scenarios, uniformly sampling the GD order is not efficient. We have recently shown theoretically that optical interferometry can be modeled as a linear measurement problem and is hence subject to compressive sensing techniques that exploit the sparsity of the signal in some modal basis^52^. These findings can considerably reduce the number of measurements in the methodology presented here.

We have implemented here the GDs for the Hilbert spaces associated with HG and radial LG modes, the fFT and fHT, respectively. More generally, our approach indicates the potential utility of *yet-to-be-discovered* optical fractional transforms and provides a roadmap for their discovery. Given any modal set of interest, a fractional transform may be constructed out of the outer product of these functions in the diagonal representation given in Eq. 1 – and this fractional transform ‘delays’ the beam in its associated Hilbert space. For example, one may form a fractional transform from a basis of OAM and Bessel functions for the analysis of beams emerging from optical fibers or circular waveguides.

## Methods

### Properties of a generalized delay

Consider a functional basis 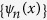 that is orthonormal 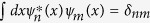 and complete 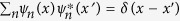. Using this set as a basis for a 1D finite-energy beam *E(x*) (in the space of square-integrable functions L^2^), we have 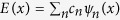, with modal coefficients 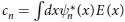. For convenience, we normalize the beam energy (the length of a vector in the Hilbert space L^2^): 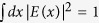; consequently, 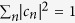.

Consider a linear, unitary transformation between input and output planes identified by coordinates *x*′ and *x*, respectively. The transformation has a real, continuous order-parameter *α* that uniquely identifies the transformation 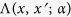. A field *E(x*) traversing this system is transformed according to Eq. (2), *E(x*) → *E(x*; *α*). Unitarity implies that 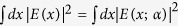 for all *α* and arbitrary *E(x*), which implies that


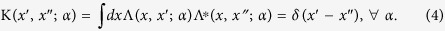


One can thus obtain the form of the GD transformation 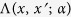 in Eq. 1, which further entails that the set of transformations 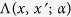 forms over *α* a one-parameter group. Defining the group composition operation as the cascade of two transformations, 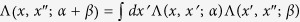, which is closed on this set, we have the requisite properties for a group: (I) the set has an identity 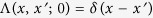; (II) the group composition operator is associative; and (III) there exists a unique inverse for any transformation 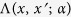, namely 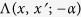. The group is also obviously commutative. Finally, the property of the inverse and the unitarity of Λ together imply that


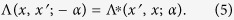


### Implementation of the 1D fFT using SLMs

The 1D fFT is defined by Eq. 1 after substituting the 1D HG functions for 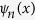. Explicitly, the 1D fFT is given by the canonical transformation


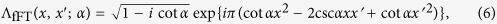


where *x* and 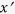 are normalized and unitless. Several specific angular orders of the fFT are readily recognizable. At *α* = 0, the system is 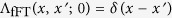, which is an imaging system without inversion or the identity operator; at 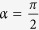, 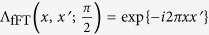 is a Fourier transform system; and at *α* = *π*, 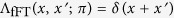, which is an imaging system with inversion.

The system in Fig. 3a consists of three cylindrical lenses (implemented by SLMs) of powers *p*_1_, *p*_2_, and *p*_1_ (inverse focal lengths) separated by equal distances *d*, and can perform the 1D fFT of arbitrary order, without scaling or additional spatially varying phase, while using the minimal number of optical components^40^. By introducing a characteristic length scale *σ* (to be set shortly) to normalize *x* and 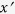, the impulse response function of this system at a wavelength *λ* is


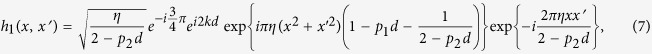


where 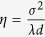 is a unitless parameter that combines all the length scales in the system. Comparing Eq. 7 to Eq. 6, we identify the lens strengths *p*_1_ and *p*_1_ that are necessary to implement the fFT of angular order *α*:


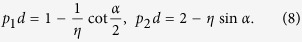


In the case of a polarization-selective SLM, the impulse response function for the H-component is Eq. 7 whereas that for the V-component corresponds to free-space propagation for a distance 2*d*.

The modified system in Fig. 3b includes two identical lenses with focal lengths *f* in addition to the three SLMs implementing cylindrical lenses with strengths *s*_1_, *s*_2_, and *s*_1_, and all the separating distances are equal to *f*. The impulse response function for the V-component is 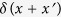, corresponding to imaging with inversion (a 4*f* imaging system). For the H-component, the impulse response function is a result of all five optical components (three when the system is folded back on itself) is given by:


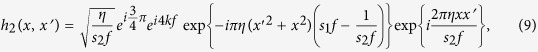


where 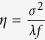 and we have introduced the transverse length scale *σ* as above. We identify 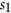 and 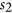 that implement the 1D fFT of order *α*


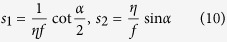


This polarization interferometer thus achieves both goals: the H-component undergoes a 1D fFT whereas the V-component reference is imaged via a 4*f* system, both without introducing extra spatial phases or scaling.

### Implementation of the radial fHT using SLMs

The 2D fFT between input plane 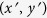 and output plane (*x, y*) is separable along the two Cartesian coordinates, such that


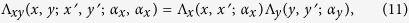


where 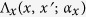 and 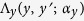 are 1D fFTs of order *α*_*x*_ (along *x*) and *α*_*y*_ (along *y*), respectively. These fFTs may be controllably accessed independently by *adding* the phase patterns for the two required crossed generalized cylindrical lenses to be implemented by the SLMs. The fHT corresponds to a symmetric 2D fFT^34^,^38^ 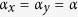. In polar coordinates we have 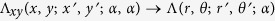. When restricted to azimuthally symmetric functions *E(r, θ*) = *E(r*), Λ itself becomes independent of *θ* and 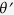, 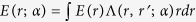, where the purely radial transformation 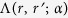 is the fHT, which is thus given by ref. 33


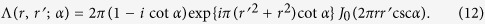


Here 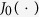 is the zeroth-order Bessel function of the first kind.

### Experimental setup

The optical beam is derived from a laser diode at a wavelength of 808 nm that is spatially filtered by coupling into a single-mode fiber at the operating wavelength (Thorlabs, FS-SN-4224) and collimated using a fiber-integrated collimation package. This produces an approximate Gaussian beam whose size is controlled by a variable beam expander (Thorlabs, BE02-05-B) moving along with the collimation package along a rail mount to yield a Gaussian beam with a FWHM of 0.6 mm located at SLM_0_. The beam is polarized along H and is modulated by SLM_0_ to produce the desired beam. The field at SLM_0_ is imaged to SLM_1_ – through a beam splitter – via a 4*f* imaging system comprised of equal-focal-length lenses (*f* = 300 mm) and the polarization is rotated from H to 45° by a half-wave plate. All the SLMs are reflection-mode, polarization-sensitive Hamamatsu LCOS-SLM (X10468-02) that modulate H but not V. The angle of incidence on SLM_1_ is less than 10°, the reflected beam passes through a lens L_1_ (*f* = 500 mm) and is normally incident on SLM_2_ reflecting back through L_1_ to SLM_1_ again. The plane of SLM_1_ is then imaged to the detector plane through the beam splitter and analyzed at +45° polarization. The image of the modified interference beam is recorded by a CCD camera (The Imaging Source, DFK 72BUC02). The SLMs are computer-controlled to synchronize the display of the phases required to implement the fFT of desired order.

## Additional Information

**How to cite this article:** Martin, L. *et al*. Basis-neutral Hilbert-space analyzers. *Sci. Rep.* **7**, 44995; doi: 10.1038/srep44995 (2017).

**Publisher's note:** Springer Nature remains neutral with regard to jurisdictional claims in published maps and institutional affiliations.

## Supplementary Material

Supplementary Information
