# Supplementary material for: Basis-neutral Hilbert-space analyzers
Source: Sci Rep. 2017 Mar 27;7:44995. doi: 10.1038/srep44995 (PMC5366812; doi:10.1038/srep44995)
Supplement: Supplementary Information [file srep44995-s1.pdf]

Basis-neutral Hilbert-space analyzers

Lane Martin[1], Davood Mardani[2], H. Esat Kondakci[1], Walker D. Larson[1], Soroush Shabahang[1], Ali K. Jahromi[1], Tanya Malhotra[3,4], A. Nick Vamivakas[4,5], George K. Atia[2], and Ayman F. Abouraddy[1*]

[1]*CREOL, The College of Optics & Photonics, University of Central Florida, Orlando, FL 32816, USA*

[2]*Dept. Electrical Engineering and Computer Engineering, University of Central Florida, Orlando, FL 32816, USA*

[3]*Department of Physics and Astronomy, University of Rochester, Rochester, New York 14627, USA*

[4]*Center for Coherence and Quantum Optics, University of Rochester, Rochester, New York 14627, USA*

[5]*Institute of Optics, University of Rochester, Rochester, NY 14627, USA*

*Corresponding author; email: raddy@creol.ucf.edu

Supplementary Information

In this *Supplementary Information* document, we provide additional details on the experimental setup and the simulations presented in the main text. Most importantly, we assess the impact of the hardware limitations and physical constraints imposed on the optical components on the performance of our modal analysis scheme; particularly the spatial light modulators (SLMs) used in the generalized interferometer (Fig. 3 of the main text). We pay special attention to the imperfections observed in the neighborhood of the fractional transforms midpoints ($\alpha = 2$ for the fractional Fourier transform). In pursuit of this goal, we provide simulations using 1D Hermite-Gaussian (HG) modes and their superpositions while examining the three main hardware constraints imposed on the SLMs: (1) the active area of the SLM chip; (2) the SLM pixel size; and (3) the number of quantized phase levels. We investigate the effects of these limitations on the errors resulting from making use of the interferometric measurements in modal reconstruction.

S1. Details of the experimental setup

To implement the fractional Fourier Transform (fFT), we use three electrically addressable SLMs. All the SLMs are reflection-mode, polarization-sensitive, Hamamatsu LCOS-SLM (X10468-02) with the specifications given in Table 1. A detailed schematic for the experimental layout is given in Fig. S1. The lenses with focal distances $f = 500$ mm are used in the experiment in $2f$-configuration.

Table S1 | Physical specifications of the SLM

DVI signal format	SVGA (800×600 pixels)
Number of quantized phase levels	256 levels (8 bits)
Effective area	16×12 mm

S2. Simulation model

In our simulations, we model the SLMs as thin phase screens imparting the following distributions to the incident field,

$$\Phi_1(x) = \Phi_3(x) = \exp\left\{-i\frac{\pi p_1 x^2}{\lambda}\right\}, \quad \text{(S1)}$$

$$\Phi_2(x) = \exp\left\{-i\frac{\pi p_2 x^2}{\lambda}\right\}, \quad \text{(S2)}$$

where $\Phi_j(x)$ denotes the phase used for j^{th} SLM, $j = 1,2,3$. To select an fFT of order α, we use the corresponding values of p_1 and p_2 given in the main text (Methods). We use Fresnel integrals to model diffraction and the lenses between the consecutive SLMs.

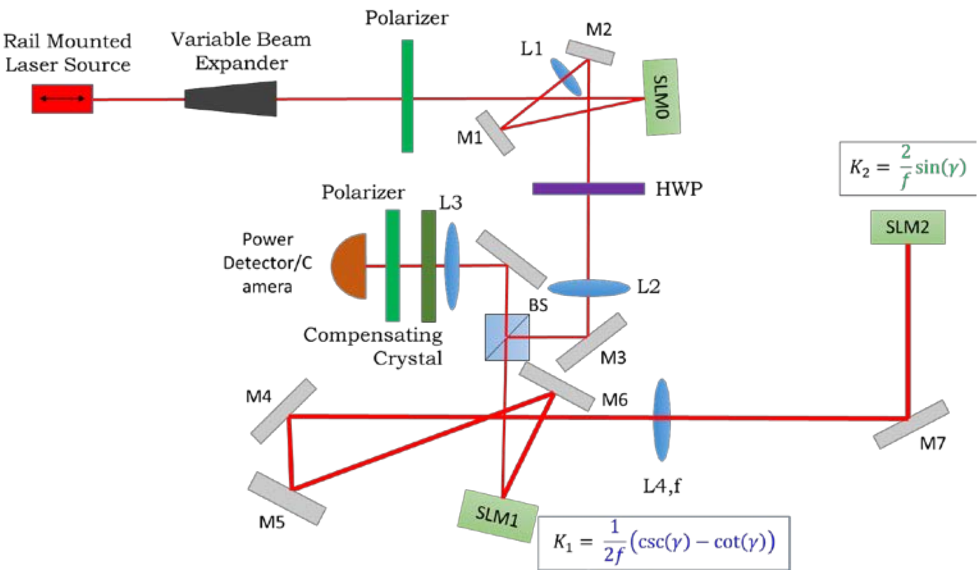

Figure S1 | Experimental setup. L1 through L4 are lenses; SLM0 through SLM2 are spatial light modulators; BS: beam splitter; HWP: half-wave plate; M1 through M7 are mirrors. K_1 and K_2 are the strengths of the lenses implemented on SLM1 and SLM2 (Methods in the main text); f is the focal length of lens L4; γ is the fFT order.

We consider the effects of three physical constraints of the SLMs in our simulations:

1. *SLM size*: The physical dimension of the active area on each SLM chip along the x coordinate is 16 mm. To capture this effect in our simulations, we use a rectangular window of width 16 mm to restrict the beam along the x coordinate and set the field outside it to zero. The SLM effectively clips an incident beam along x.

2. *SLM pixel size*: Each SLM along x has 800 pixels of size 20 μm each. The phase imparted by the SLMs is piecewise constant along 20 μm intervals. In the simulations, we sample the phase with a sampling period of 20 μm to capture this effect.

3. *The number of quantized phase levels*: The SLM phase does not assume arbitrary values between 0 and 2π; instead, only 256 levels are accessible. We approximate the target phase values, given in Eq. S1 and Eq. S2, by selecting the closest quantized phase level.

S3. Optical beams used to approximate the Hermite-Gaussian and Laguerre-Gaussian modes

The input beams we used in our experiments and simulations are approximate forms of the exact Hermite-Gaussian (HG) and Laguerre-Gaussian (LG) modes. They are obtained from an initial Gaussian beam $\mathrm{HG}_0(x)$ through phase-modulation implemented by SLM0 in Fig. S1. The approximate mode $\mathrm{HG}_1(x)$ is obtained by shifting the phase by π for $x < 0$. We follow this procedure to produce the higher-order modes HG_2 and HG_3. The approximate HG beams are plotted in Fig. S8. Azimuthally symmetric phase-modulation is implemented in the radial direction to obtain approximate LG_1 and LG_2 modes form the Gaussian LG_0 mode, as shown in Fig. S9. These approximations of the pure HG and LG modes consequently have contributions (non-vanishing projections) to other HG and LG modes in their modal reconstruction.

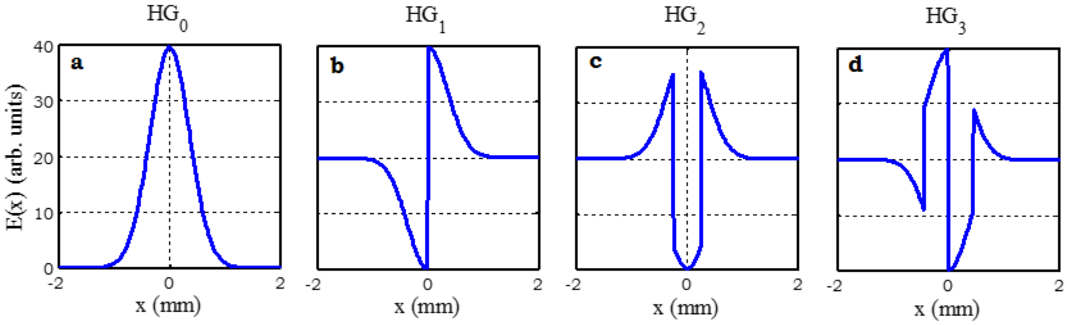

Figure S2 | Approximate Hermite-Gaussian (HG) beams produced from a Gaussian beam via phase-modulation. **a**, HG_0. **b**, HG_1. **c**, HG_2. **d**, HG_3.

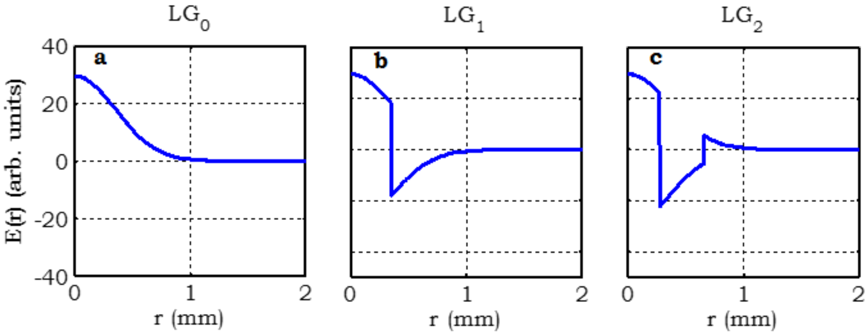

Figure S3 | Approximate Laguerre-Gaussian (LG) beams produced from a Gaussian beam via phase-modulation. **a**, LG_0. **b**, LG_1. **c**, LG_2.

S4. Simulations of modal reconstruction for ideal experimental configuration

Figure S4 through Fig. S6 depict fractional transforms and 2D interferograms for individual HG modes, individual LG modes, and superposed HG modes, corresponding to the results in Fig. 4 through Fig. 6, respectively, in the main text. These calculations do not take into consideration the imperfections of the SLMs sued in the experiment.

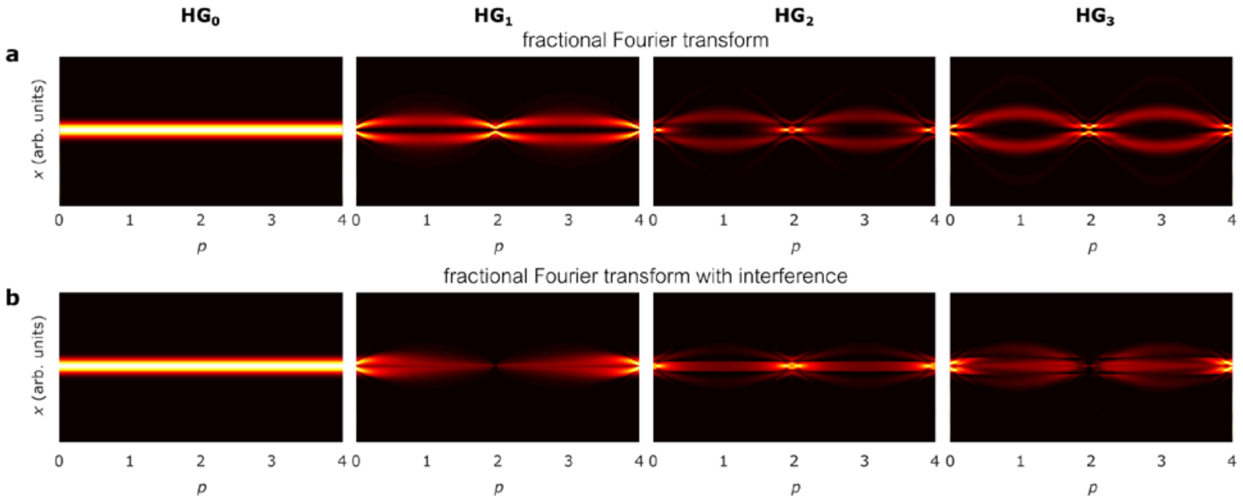

Figure S4 | Numerical simulation of fractional Fourier transform of Hermite-Gaussian modes. **a**, The fFT without interference with a reference beam. **b**, Results of interfering the fFT with a reference beam. From left to right, we plot results for the (approximate) HG_0, HG_1, HG_2, and HG_3 beams.

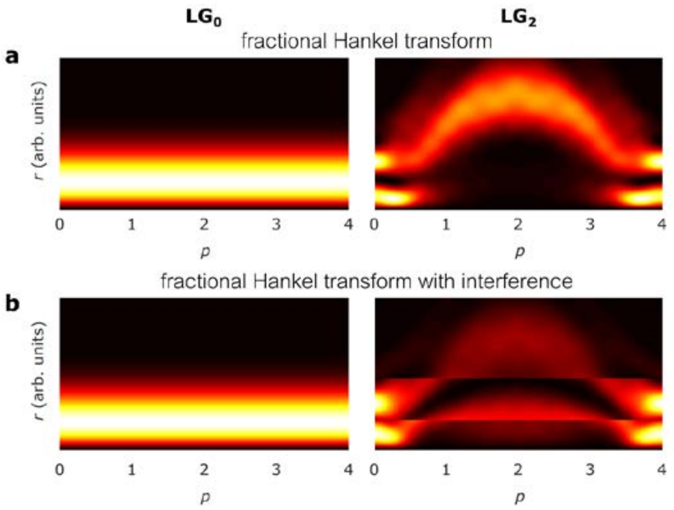

Figure S5 | Numerical simulation of fractional Hankel transform of Laguerre-Gaussian modes. **a**, The fHT without interference with a reference beam. **b**, Results of interfering the fHT with a reference beam.

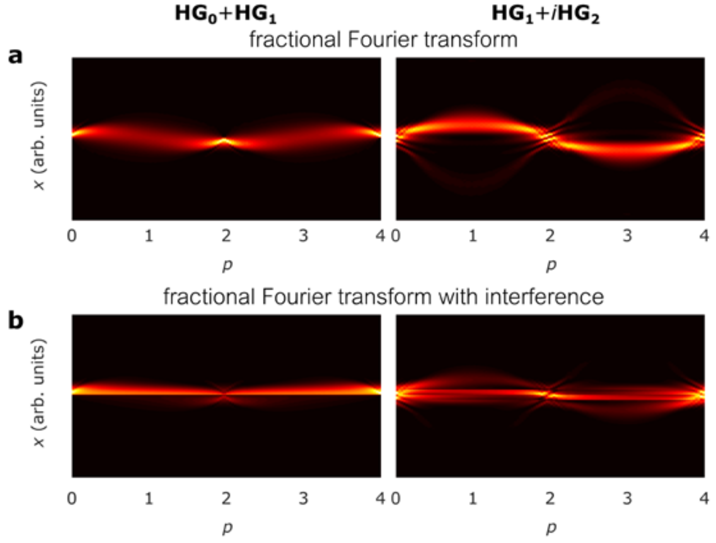

Figure S6 | Numerical simulation of fractional Fourier transform of superposed HG modes. a, The fFT without interference with a reference beam. **b**, Results of interfering the fFT with a reference beam. On the left we plot the results for $\mathrm{HG}_0 + \mathrm{HG}_1$ and on the right for $\mathrm{HG}_1 + i\mathrm{HG}_2$.

S5. Impact of SLM imperfection on modal reconstruction

Using the mathematical model described above, we study the effects of SLM size (which results in clipping of the beam), SLM pixel size, and phase-quantization on the system performance. Let $\hat{\boldsymbol{c}}$ denote the reconstructed vector of modal energies, i.e., its ith entry is the energy of the ith mode. To assess the performance of the system with respect to the three limiting factors, we define a reconstruction error

$$\text{reconstruction error} \equiv \|\boldsymbol{c} - \hat{\boldsymbol{c}}\|_2^2, \tag{S3}$$

where $\boldsymbol{c}$ is a vector containing the true modal energies of the input beam. To study the effect of the physical constraints, we reconstruct $\hat{\boldsymbol{c}}$ and calculate the reconstruction error for different values of SLM size, pixel size, and number of the quantized phase levels. Figures S7-S9 illustrate a sample from these results.

We consider the input beams to be the four lowest order HG modes: HG_0, HG_1, HG_2, and HG_3. Figure S7 depicts the effect of the SLM size. We find that an SLM size of 16 mm is appropriate for modal analysis. To separate out the effect of pixel size on modal reconstruction, we have set it to 10 μm. Figure S8 shows the reconstruction error versus the pixel size (SLM size set to 16 mm). The reconstruction error saturates (around zero) when the pixel size $\leq$ 10 μm. With the physical pixel size of 20 μm of the used

hardware, there is a graceful degradation in the performance of modal reconstruction. Figure S9 shows the effect of the number of phase quantization levels on the reconstruction quality with an SLM size of 16 mm and pixel size of 10 μm. The reconstruction error for all the modes drops to zero as long as the number of quantized phase levels is ≥ 100. Therefore, we expect the effect of quantization on modal reconstruction to be negligible with the 256 input SLM phase levels.

In Fig. S10 through Fig. S12, we plot the intensity distribution after interfering the fFT beam with the original beam as a reference, the interferogram $I(p)$, and the Fourier transform of the interferogram, which constitutes the modal reconstruction. Figure S10 presents the results for HG_2, Fig. S11 for HG_3, and Fig. S12 for $\mathrm{HG}_0 + \mathrm{HG}_1$. For each beam, we repeat the simulations under three sets of conditions: (1) only a finite SLM size of 16 mm; (2) finite SLM size of 16 mm and finite pixel size of 20 μm; (3) the combined effect of a finite SLM size of 16 mm, a finite pixel size of 20 μm, and 256 quantized phase levels. The effects of these imperfections on the simulations match those observed in our data reported in the main text.

An anomaly is seen in the interferograms at $p = 2$ in the form of a dip that is observed in both the experimental results and the simulations. This anomaly is due to the hardware limitations, particularly the SLM size that results in beam clipping. To confirm this, we repeat the simulations for the HG_2 beam for different hardware specifications. In Fig. S15a, we set the SLM size to 16 mm and pixel size to 10 μm. A drop in the interferogram at $p = 2$ is observed. By decreasing the pixel size to 5 μm, the dip at $p = 2$ persists (Fig. S8b). This suggests that the pixilation of the SLMs is not related to the observed drop at $p = 2$. In Fig. S8c, we increase the SLM size to 60 mm and observe that the dip at $p = 2$ is eliminated. These results indicate that the SLM size is likely the main limitation set be the hardware on modal reconstruction.

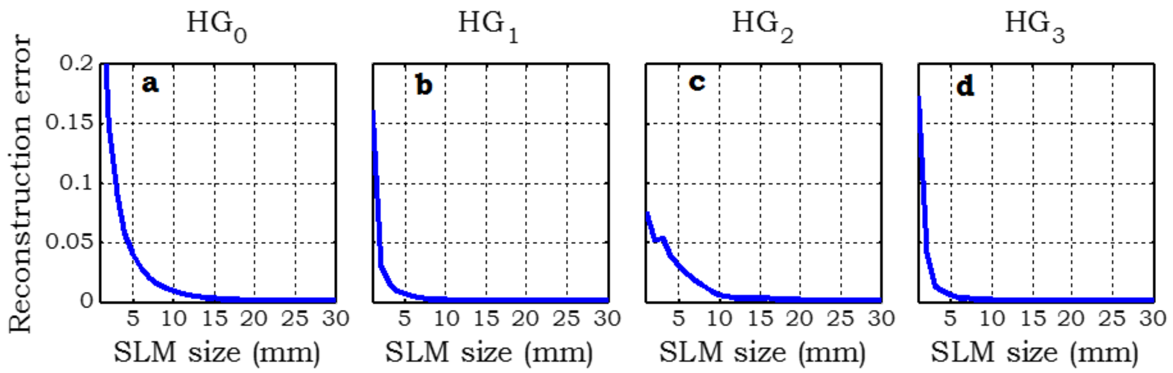

Figure S7 | Reconstruction error versus SLM size. a, HG_0. **b**, , HG_1. **c**, , HG_2. **d**, , HG_3. The pixel size 10 µm and the number of quantized phase levels is 256.

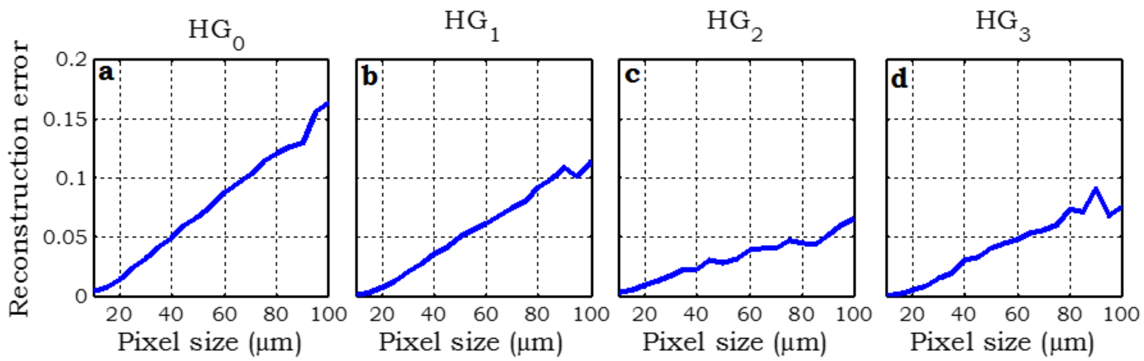

Figure S8 | Reconstruction error versus the pixel size. a, HG_0. **b**, HG_1. **c**, HG_2. **d**, HG_3. The SLM size is 16 mm and the number of SLM quantized phase levels is 256.

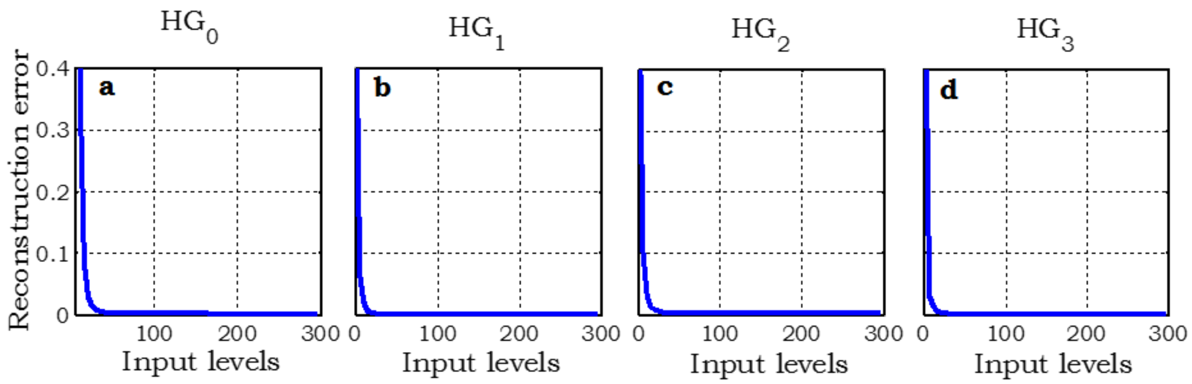

Figure S9 | Reconstruction error versus the number of quantized phase levels. a, HG_0. **b**, HG_1. **c**, HG_2. **d**, HG_3. The SLM size is 16 mm and the pixel size is 10 µm.

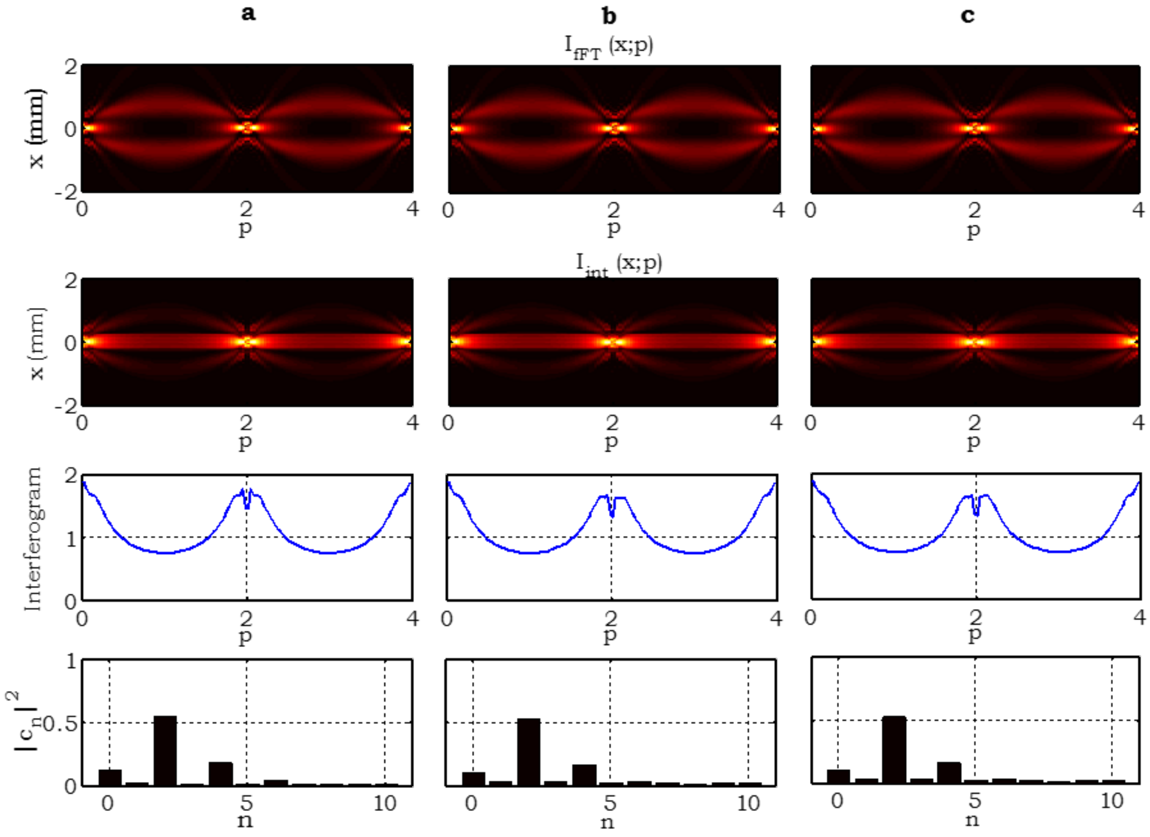

Figure S10 | HG_2-Modal analysis using generalized optical interferometry. **a**, Considering a finite SLM size of 16 mm. **b**, Considering a finite SLM size of 16 mm and a pixel size of 20 µm. **c**, Considering a finite SLM size of 16 mm, a pixel size of 20 µm, and 256 quantized phase levels. First row shows the fFT, second row shows the interference of the fFT with the reference beam, the third row shows the interferogram, and the fourth row the reconstructed coefficients.

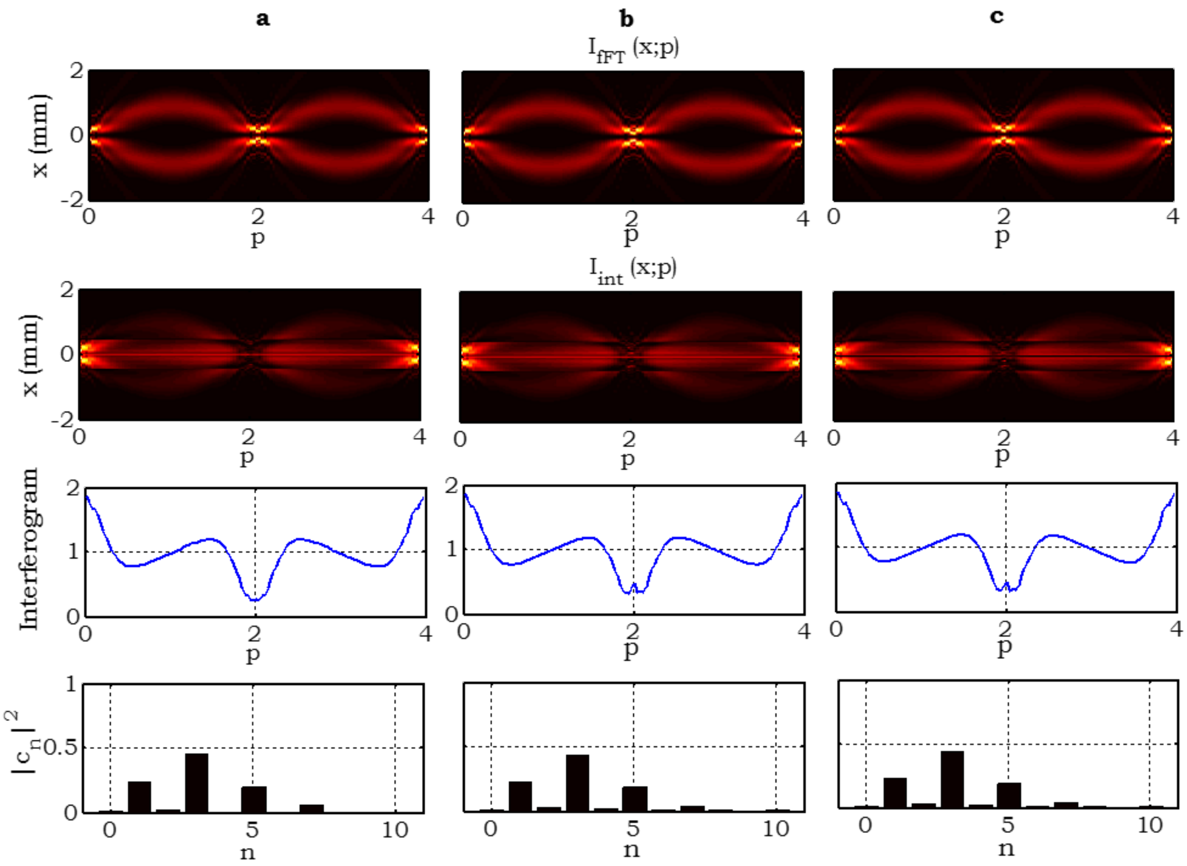

Figure S11 | HG_3-Modal analysis using generalized optical interferometry. a-c, Same as (**a-c**) in Fig. S12.

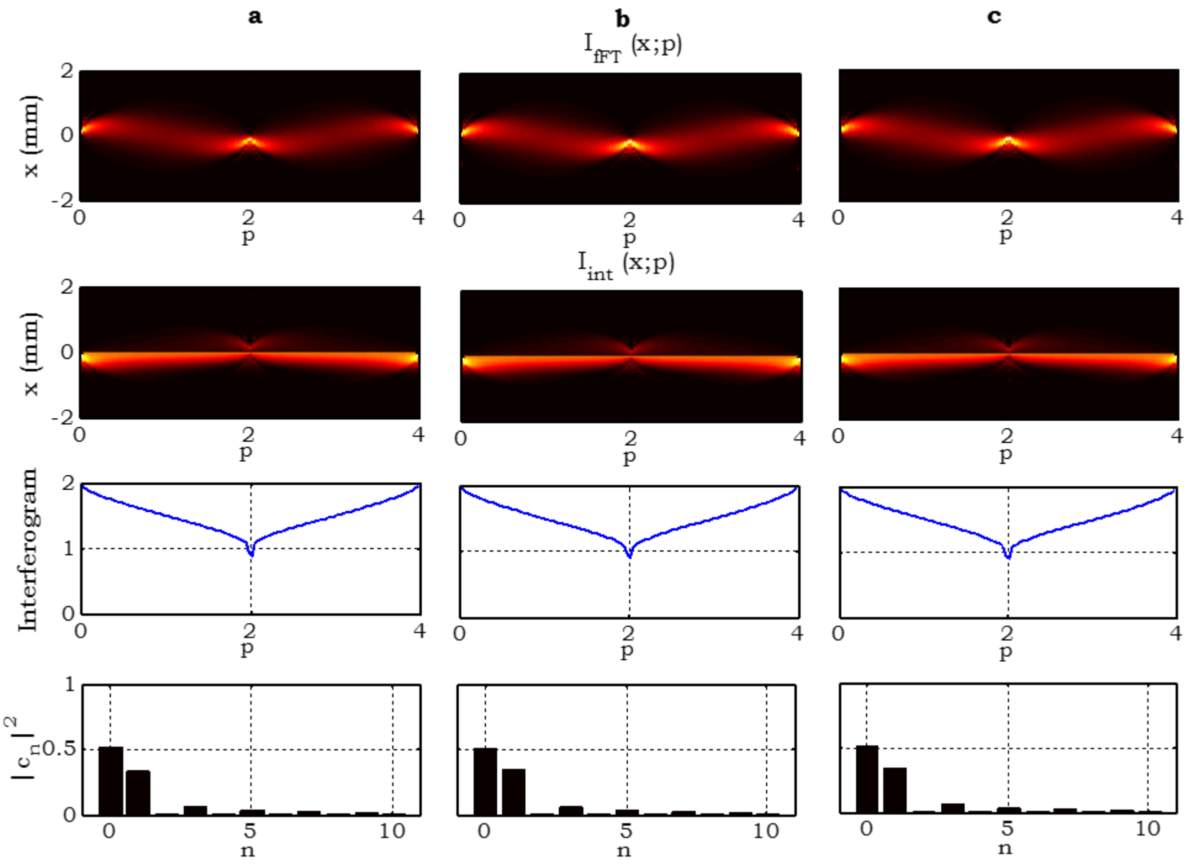

Figure S12 | HG_0 + HG_1-Modal analysis using generalized optical interferometry. **a-c**, Same as (**a-c**) in Fig. S12.

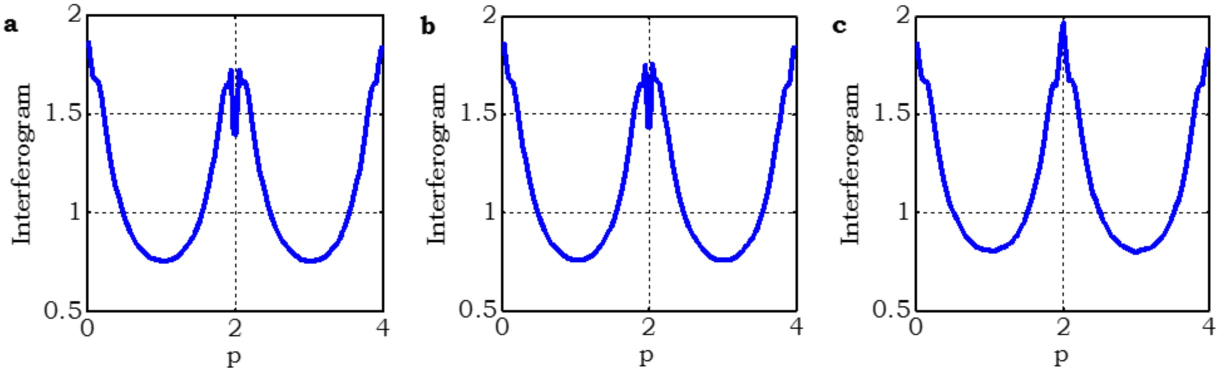

Figure S13 | Investigating the effect of the SLM limitations of the recorded interferogram produced by the HG_2 mode. a, SLM size is 16 mm and pixel size is 10 µm. **b**, The SLM size is 16 mm and the pixel size is 5 µm. **c**, The SLM size is 60 mm and the pixel size is 10 µm.
